# Achalasia: Physician Practices and Knowledge in Türkiye

**DOI:** 10.5152/tjg.2026.25713

**Published:** 2026-01-19

**Authors:** Ayça Eroğlu Haktanır, Altay Çelebi

**Affiliations:** Department of Gastroenterology, Kocaeli University Faculty of Medicine, Kocaeli, Türkiye

**Keywords:** Achalasia, diagnostic delay, esophageal motility disorder, high-resolution manometry, physician awareness, Türkiye

## Abstract

**Background/Aims:**

Achalasia is a rare esophageal motility disorder often underrecognized due to nonspecific symptoms and limited physician awareness. Although diagnostic tools have advanced, delays remain common. Previous studies in Türkiye were mainly single-center or review-based, with no nationwide assessment of physician-related factors. This study evaluated physician knowledge, diagnostic practices, high-resolution esophageal manometry (HREM) access, and factors influencing diagnostic delay, providing the first nationwide achalasia-focused dataset.

**Materials and Methods:**

A web-based survey was conducted among 4216 physicians; 675 responses (16.0%) were analyzed. The 32-item questionnaire included demographics, achalasia knowledge, diagnostic/referral practices, HREM accessibility, and training. Participants included 12.3% primary care physicians, 26.4% secondary-level, 26.2% tertiary training/research, and 35.1% university hospital physicians. Overall, 89.6% practiced internal medicine, 9.8% surgical sciences, and 0.6% basic medical sciences.

**Results:**

Male physicians demonstrated higher knowledge (60.8% vs. 39.2%; *P* < .001) and diagnostic recognition, whereas females reported more self-perceived deficiencies (*P* < .001). Gastroenterologists had superior diagnostic accuracy (*P* < .001), but easy HREM access was limited (9.1%). Physicians in tertiary hospitals showed higher knowledge and diagnostic accuracy (*P* = .025 and *P* = .040). Participation in training programs and treatment familiarity did not vary by hospital type (*P* = .437 and *P* = .512).

**Conclusions:**

Variations in physician knowledge and diagnostic practices across specialties, hospital types, and gender may contribute to delayed achalasia recognition. Persistent gaps in practical competence, HREM familiarity, and access to diagnostic resources highlight the need for targeted education and structured interventions. Improving diagnostic infrastructure and HREM access may enable earlier diagnosis and enhance outcomes.

**Main Points**

- Physician knowledge and diagnostic approaches to achalasia show significant variation across specialties, hospital settings, and gender in Türkiye.
- Restricted access to high-resolution esophageal manometry (HREM) and insufficient formal education remain the leading causes of diagnostic delay.
- Gastroenterologists and internists exhibit superior diagnostic accuracy and referral practices compared with surgeons and non-specialists.
- Nationwide standardized training and broader HREM availability are essential to enhance early recognition and improve patient outcomes.
- Disparities related to gender and institutional background underscore the importance of equitable education and structured interdisciplinary collaboration.

## Introduction

Achalasia is a rare primary esophageal motility disorder characterized by impaired relaxation of the lower esophageal sphincter and loss of coordinated peristalsis, presenting clinically with progressive dysphagia, regurgitation, retrosternal discomfort, and unintentional weight loss.^1^^-^^4^ Despite its low incidence (1-3 per 100 000 annually), achalasia substantially reduces quality of life and can lead to serious complications, including aspiration pneumonia, esophageal dilation, malnutrition, and, in advanced cases, increased risk of esophageal carcinoma.^3^^,^^5^ The progressive nature of the disorder underscores the importance of timely diagnosis and intervention.^5^ However, even in centers with advanced diagnostic capabilities, diagnostic delay remains a critical issue. In a large single-center series of 278 patients diagnosed between 2013 and 2023, the median time from symptom onset to definitive diagnosis was 24 months (2-72), and more than three-quarters of the patients experienced a delay >12 months.^6^ Similarly, in a multicenter survey from Germany, median diagnostic delay was 25 months (9-65), with many patients seeing 3 or more specialists before manometry was finally performed—only about 70% of patients underwent the gold-standard test before diagnosis.^7^ Early recognition is challenging due to nonspecific symptoms that overlap with common gastrointestinal disorders such as gastroesophageal reflux disease and functional dyspepsia,^6^^-^^10^ often resulting in misdiagnosis, repeated investigations, and inappropriate treatment. While patient-related factors, such as symptom reporting, contribute to diagnostic delay,^5^^-^^7^^,^^11^ physician-level determinants—including knowledge gaps, clinical suspicion, and familiarity with diagnostic modalities—play a critical role in prolonging the interval from symptom onset to confirmed diagnosis.

High-resolution esophageal manometry (HREM) is the gold standard for achalasia diagnosis, yet awareness and appropriate utilization remain inconsistent.^12^^,^^13^ Limited familiarity contributes to delayed recognition, suboptimal referral, and inappropriate diagnostic testing, highlighting the need for structured education, guideline dissemination, and targeted training. Existing surveys on gastrointestinal motility disorders or trainee exposure to neurogastroenterology focus primarily on broad motility concepts rather than achalasia-specific challenges, and most have been conducted outside Türkiye.^14^^,^^15^

In Türkiye, healthcare is organized across primary, secondary, and tertiary levels, with heterogeneity in infrastructure, physician training, and access to specialized diagnostics.^6^^,^^11^^,^^12^ Tertiary centers generally provide HREM capability, whereas lower-level hospitals often face limitations in equipment, personnel, and formal training, potentially delaying referral and diagnosis.

To the best of knowledge, while several small case series of achalasia patients have been reported from Türkiye, there is no nationwide study systematically evaluating physician-level knowledge, diagnostic practices, referral behaviors, and educational exposure across different specialties and levels of health-care delivery—a significant unmet need.

This survey was designed to evaluate physician knowledge, diagnostic approaches, referral patterns, and prior educational exposure across specialties and healthcare settings. Identifying gaps and barriers in clinical practice can inform targeted educational initiatives, standardize training, and guide resource allocation to promote timely recognition and management, while considering gender- and institution-related differences to ensure equitable physician training and optimize patient outcomes.

## Materials and Methods

This cross-sectional study employed an anonymous, web-based questionnaire to assess physicians’ knowledge, diagnostic practices, and perceptions regarding achalasia, as well as access to HREM across Türkiye. Participation was voluntary, and no personal identifiers were collected to ensure confidentiality. Eligible participants included physicians working in primary, secondary, and tertiary care facilities, including public and university hospitals.

In Türkiye, medical education consists of a 6-year undergraduate program followed by residency training in a chosen specialty, with optional subspecialty training available. This structured training ensures baseline competencies while exposure to gastroenterology—and consequently achalasia awareness—varies by specialty and institutional affiliation.

Physicians were categorized into 3 groups: primary care physicians, internal medicine disciplines, and surgical sciences. Recruitment was conducted through professional networks, and the survey link was distributed via Google Forms and shared in professional WhatsApp groups. Of 4216 invited physicians, 675 (16.0%) completed the survey.

The primary outcomes of this study were defined as physicians’ correct identification of HREM as the gold-standard diagnostic test for achalasia, frequency of inquiry about dysphagia, self-reported adequacy of knowledge regarding achalasia treatment options, and reported accessibility of HREM in their institutions.

### Questionnaire

The study-specific, investigator-developed questionnaire was based on a literature review and expert input from gastroenterologists. Although not formally validated or piloted, items were reviewed and refined by the research team to ensure clarity and relevance. A self-administered, 32-item questionnaire evaluated 4 domains:

1. Demographics: Age, gender, specialty, institutional affiliation (university, training and research, state, private hospitals, primary care), and professional status (resident, specialist, consultant).
2. Knowledge and awareness: Familiarity with achalasia; frequency of symptom inquiry (dysphagia, regurgitation, chest pain, weight loss, and cough); recognition of key clinical signs and differential diagnoses; and awareness of diagnostic tools (barium swallow, endoscopy, HREM, computed tomography, and magnetic resonance imaging). Participants were specifically asked to identify the gold-standard diagnostic test (HREM), which served as one of the primary outcomes. Respondents not selecting HREM were classified as “participants unaware” of the correct diagnostic method.
3. Diagnostic and referral practices: Number of cases diagnosed/referred, referral patterns, perceived diagnostic delays and causes, HREM accessibility, and patient-related delays. The HREM accessibility was defined as a primary outcome.
4. Education and awareness strategies: Adequacy of training, perceived knowledge gaps regarding treatment options (endoscopic balloon dilation, Heller myotomy, peroral endoscopic myotomy (POEM)), and evaluation of awareness strategies including continuing medical education (CME), congress presentations, guidelines, case discussions, social media, and patient resources. Self-reported adequacy of treatment knowledge was included as a primary outcome.

### Data Collection

The survey was administered online via Google Forms, ensuring 1 submission per device to prevent duplication. Electronic informed consent was obtained from all participants prior to survey initiation.

### Ethical Considerations

Ethical approval was obtained from the Non-Interventional Clinical Research Ethics Committee of Kocaeli University (Decision No: 2025/15/26; Project No: 2023/379) on July 3, 2025, and the study adhered to the Declaration of Helsinki.

### Statistical Analysis

Responses were securely stored and analyzed using IBM SPSS Statistics version 29.0 (IBM SPSS Corp.; Armonk, NY, USA). Categorical variables were presented as frequencies and percentages. Univariate analyses were performed using chi-square tests to explore crude associations between categorical variables. These tests were considered exploratory, and therefore no multiple testing correction (e.g., Bonferroni) was applied. To identify independent predictors, multivariable logistic regression models were constructed for both achalasia knowledge and HREM familiarity, and results were reported as odds ratios (ORs) with 95% CIs. A *P* value < .05 was considered statistically significant.

## Results

### Participant Characteristics

A total of 675 physicians completed the survey. Most were from the Marmara region (65.0%, n = 420), followed by Central Anatolia and Aegean (7.7% each, n = 38), Black Sea (7.1%, n = 35), Mediterranean (6.5%, n = 32), Eastern Anatolia (6.5%, n = 32), and Southeastern Anatolia (4.1%, n = 23). The study cohort primarily consisted of physicians aged 35-44 years (39.1%), followed by those aged 24-34 years (27.6%), 45-54 years (22.8%), and 55 years or older (10.5%), indicating a predominance of mid-career participants. Female physicians comprised 57.5% (n = 388). Gastroenterologists accounted for 9.8% (n = 66), while 90.2% (n = 609) were non-gastroenterology specialists. Primary care physicians accounted for 12.3% of participants. By discipline, 89.6% practiced internal medicine disciplines, 9.8% surgical sciences, and 0.6% basic medical sciences.

Professional experience varied: ≤2 years, 8.0%; 3-5 years, 19.3%; 6-10 years, 10.5%; 11-15 years, 19.6%; ≥16 years, 34.9%. Of the respondents, 12.3% were employed in primary care centers, 26.4% in secondary-level hospitals, 26.2% in tertiary training and research hospitals, and 35.1% in tertiary university hospitals, indicating a balanced representation across different healthcare levels (Table 1).

### Knowledge and Awareness of Achalasia

While 75.4% of participants self-reported adequate knowledge of achalasia, 78.1% simultaneously acknowledged gaps, indicating limited confidence in clinical recognition. Only 21.8% routinely inquired about dysphagia, regurgitation, or chest pain, whereas over half did so rarely or never. Regarding familiarity with HREM, 79.7% of respondents reported being knowledgeable about the technique, whereas 20.3% indicated they were not familiar with it. Knowledge gaps were most evident in diagnostic differentiation, with 31.4% of participants classified as unaware for selecting incorrect diagnostic modalities. This indicates that awareness of HREM does not necessarily translate into its practical implementation (Table 2).

### Referral Patterns and Diagnostic Accessibility

Regarding patient referral for achalasia, 44.0% of respondents had referred at least 1 patient, while 56.0% reported never having referred a patient for further evaluation or management. Structural barriers were significant: only 1.6% reported easy access to HREM, 59.4% found access difficult, and 5.6% considered it completely unavailable. One-third (33.3%) were uncertain about local HREM availability, reflecting institutional variability. Physicians identified lack of awareness (66.6%) as the leading cause of diagnostic delay, followed by patient noncompliance (47.0%), late presentation (46.6%), and restricted access to diagnostic tools (44.0%). Additional contributing factors included high patient volume, nonspecific symptoms, misattribution of symptoms, scarcity of manometry centers, and fragmented referral pathways.

### Educational Exposure and Treatment Awareness

Regarding formal achalasia education, 45.2% of respondents reported having received it, 49.8% indicated partial exposure, and 5.0% reported no formal training. Nevertheless, 86.4% were familiar with treatment modalities such as pneumatic dilation, Heller myotomy, and POEM. Most participants (81.0%) agreed that primary care physicians require greater awareness of achalasia. Preferred educational strategies included case-based discussions (68.0%), evidence-based guidelines (60.9%), CME/webinars (54.7%), and patient-education materials (51.0%). Undergraduate education was perceived as partial by nearly half (49.3%) and sufficient by 45.7%, suggesting unmet training needs in early medical curricula (Table 2).

### Age-Related Comparisons

When assessed by age, the distribution of achalasia knowledge among respondents revealed that complete lack of knowledge was rare across all age groups (1.0% overall). The majority of participants reported having full knowledge, with the highest proportion observed in the 35-44 age group (77.7%), followed by 24-34 (75.3%), 45-54 (73.4%), and ≥55 years (71.8%). Partial knowledge was reported by 23.6% of respondents, distributed relatively evenly across age categories. Statistical analysis indicated no significant association between age and achalasia knowledge (*P* = .784), suggesting that awareness is generally moderate to high among physicians regardless of age. The distribution of HREM familiarity across age groups revealed that 79.7% of respondents reported being familiar with HREM, whereas 20.3% indicated lack of knowledge. Familiarity was highest among physicians aged 35-44 years (83.3%) and lowest in the ≥55 age group (70.4%). No statistically significant association was observed between age and HREM knowledge (*P* = .106).

### Comparisons by Gender, Specialty, and Discipline

Gender differences: Male physicians had higher rates of adequate knowledge about achalasia (81.2% vs. 71.1%; *P* < .001), more frequent symptom inquiry, and greater recognition of appropriate diagnostic methods, including HREM (*P* = .033). Female physicians more often reported self-perceived deficiencies (85.1% vs. 68.6%; *P* < .001) and uncertainty regarding HREM availability (*P* = .004). Training exposure was similar between genders (*P* = .619), and treatment awareness was marginally higher in males (*P* = .041) (Supplementary Table 1).

### Specialty Differences

Interestingly, a higher proportion of gastroenterologists reported having no formal training in achalasia compared to other specialties (12.1% vs. 4.3%; *P* = .013, intergroup *P* = .006), despite overall superior knowledge and clinical practice. This suggests that, even in the absence of formal training, gastroenterologists may acquire expertise through clinical exposure and experience. Consistently, gastroenterologists demonstrated higher achalasia knowledge (95.5% vs. 73.2%; *P* < .001) and more frequent symptom inquiry (80.3% vs. 15.4%; *P* < .001). All gastroenterologists correctly identified HREM as the gold-standard diagnostic test, whereas nearly one-third of non-gastroenterologists misclassified alternative modalities. Gastroenterologists also reported greater familiarity with treatment (*P* < .001). Despite this expertise, only 9.1% reported easy access to HREM, highlighting systemic rather than educational barriers to optimal care.

### Discipline Differences

Internal medicine specialists demonstrated higher awareness of achalasia diagnostic methods than surgical sciences (66.7% vs. 81.1%; *P* = .009). However, both groups showed similar levels of formal training exposure (*P* > .05). Additionally, internal medicine physicians more frequently recognized appropriate treatment approaches (87.2% vs. 78.8%; *P* = .089), although this difference did not reach statistical significance (Supplementary Table 1).

### Diagnostic Knowledge and Self-Perceived Competence

All gastroenterologists correctly identified the diagnostic methods, whereas 34.8% of non-gastroenterologists were unaware of them (*P* < .001). Likewise, 84.7% of non-gastroenterologists reported insufficient knowledge, compared with only 16.7% of gastroenterologists (*P* < .001), indicating both objective and self-perceived knowledge gaps among non-specialists (Supplementary Table 1).

### Impact of Hospital Level

Physicians working in tertiary institutions (both training and research hospitals and university hospitals) demonstrated significantly higher achalasia knowledge compared with primary-level physicians (*P* = .025). Intergroup comparisons showed that primary-care physicians had lower knowledge scores than those in tertiary training and research (T&R) hospitals (*P* = .027) and tertiary university hospitals (*P* = .029).

Self-perceived knowledge deficiency was markedly higher among primary-care physicians (92.8%), significantly exceeding that of secondary, tertiary T&R, and tertiary university physicians for comparisons, respectively (*P* = .005, *P* = .020, *P* = .003). Awareness of HREM showed a similar pattern, with primary-care physicians demonstrating significantly lower awareness compared with tertiary T&R and tertiary university physicians for comparisons, respectively (*P* = .005, *P* = .010) (Supplementary Table 2).

### Education and Training by Hospital Level

No significant differences were observed in achalasia training participation across hospital levels (*P* = .437), with similar proportions of physicians reporting full, partial, or no training. Similarly, knowledge of achalasia treatment was comparable across all hospital levels (83%-88%, *P* = .512), indicating uniform exposure to training and treatment information in this cohort (Supplementary Table 2).

Multivariable logistic regression identified specialty and hospital level as independent predictors of achalasia and HREM understanding. Male physicians were more likely to demonstrate adequate knowledge than females (OR = 1.63; 95% CI: 1.10-2.41; *P* = .015). Gastroenterologists exhibited substantially higher odds compared with other specialties (OR = 5.73; 95% CI: 1.75-18.83; *P* = .004), and physicians practicing in internal medicine disciplines showed greater familiarity than those in surgical sciences (OR = 1.87; 95% CI: 1.06-3.30; *P* = .032). Hospital level also influenced understanding: tertiary teaching and research hospitals (OR = 2.09; 95% CI: 1.16-3.76; *P* = .014) and tertiary university hospitals (OR = 1.87; 95% CI: 1.08-3.26; *P* = .026) outperformed primary care hospitals, while secondary hospitals did not differ significantly (OR = 1.27; 95% CI: 0.72-2.25; *P* = .402). For HREM, gastroenterology specialty remained the strongest predictor (OR = 13.30; 95% CI: 1.81-97.75; *P* = .011), with internal medicine physicians also demonstrating higher familiarity than those in surgical sciences (OR = 2.25; 95% CI: 1.25-4.04; *P* = .007). Hospital effects were consistent, with tertiary teaching and research (OR = 2.49; 95% CI: 1.35-4.58; *P* = .003) and tertiary university hospitals (OR = 2.09; 95% CI: 1.19-3.68; *P* = .011) exceeding primary care, and secondary hospitals showing moderate increases (OR = 2.04; 95% CI: 1.12-3.71; *P* = .020). Gender showed a borderline effect (OR = 1.46; 95% CI: 0.96-2.21; *P* = .076). These results indicate that both specialty and hospital level are major determinants of achalasia and HREM familiarity, with gastroenterologists and physicians at tertiary hospitals demonstrating the greatest proficiency (Table 3).

## Discussion

This nationwide survey of 675 physicians in Türkiye provides novel insights into achalasia-related knowledge, diagnostic practices, and referral patterns. Overall awareness was moderate to high, with variations across specialty, gender, discipline, and hospital type. Gastroenterologists and internal medicine specialists showed higher diagnostic accuracy and confidence, while male physicians reported greater self-perceived competence than females. Despite familiarity with achalasia and HREM, routine symptom assessment and patient referral were suboptimal, and access to HREM was limited across healthcare settings.

Previous studies on achalasia have primarily emphasized patient-related contributors—such as misinterpreted symptoms and delayed referrals^11^^,^^16^—while physician-level determinants remain underexplored. Al Mowafy et al^14^ reported significant shortcomings in physician awareness and self-assessed competency, whereas Cohen et al^15^ identified limited exposure to neurogastroenterology and esophageal motility during internal medicine and general surgery residency training. Limited knowledge of pathophysiology and diagnostic algorithms leads primary care physicians to frequently misclassify specific motility disorders as functional gastrointestinal conditions.^6^^,^^7^^,^^16^^,^^17^ Similar findings from Asia and Egypt reveal widespread knowledge deficits, low confidence, and variability in clinical practice.^14^^,^^17^ Structured educational programs have been shown to improve proficiency in esophageal manometry and motility assessment, highlighting the value of focused training.^18^^,^^19^ Persistent gaps in undergraduate and postgraduate neurogastroenterology curricula further sustain these deficiencies and negatively affect clinical competence and patient care.^18^^-^^21^

The findings address a key gap in the literature: although motility-related surveys have documented global deficiencies in neurogastroenterology education, few have evaluated achalasia-specific diagnostic decision-making or referral patterns at a national level, and data from Türkiye have been particularly limited. This study provides the first comprehensive evaluation in Türkiye of how physicians across specialties and healthcare settings approach diagnosis, referral, and HREM utilization, demonstrating substantial heterogeneity in knowledge, clinical practice, and access to diagnostic resources. By focusing specifically on achalasia, the results highlight persistent gaps in practical competence, familiarity with HREM, and formal training, underscoring the need for targeted educational and structural interventions.

The cohort mainly comprised mid-career physicians aged 35-44 years—consistent with prior surveys showing greater engagement in this age group^22^—and had a slight female predominance, aligning with the global feminization of the medical workforce.^23^ The high proportion of internal medicine and subspecialty physicians reflects their central role in evaluating complex gastrointestinal symptoms, as reported previously.^24^^,^^25^ Although inclusion of all healthcare levels strengthens external validity, voluntary participation may introduce selection bias. Physicians in tertiary centers demonstrated better diagnostic performance but still reported limited HREM access, suggesting infrastructural constraints; these associations should not be interpreted causally. Age was not associated with knowledge or referral patterns, differing from studies linking experience with competence.^26^^,^^27^ Since professional experience beyond age groups was not evaluated, such comparisons should be interpreted with caution. Knowledge gaps across all age groups, together with restricted HREM availability even in tertiary centers, indicate potential system-level rather than individual-level barriers.^28^^,^^29^ This interpretation remains hypothetical and requires confirmation through studies evaluating institutional workload, staffing, and diagnostic pathways.

Male physicians showed higher knowledge and diagnostic accuracy, a pattern possibly reflecting gender-related differences in self-confidence and perceived competence, as noted in previous literature.^30^^-^^34^ However, factors such as mentorship, workload distribution, and access to training were not assessed; therefore, these findings should be considered associative rather than causal. Future research incorporating detailed measures of seniority, institutional characteristics, and training history is needed to clarify underlying mechanisms and guide interventions aimed at reducing gender-related disparities in clinical competence.

Although 75.4% of participants reported adequate knowledge, 78.1% concurrently acknowledged deficiencies, reflecting the well-recognized paradox in which cognitive familiarity does not consistently translate into clinical practice^35^; moreover, the infrequent systematic assessment of achalasia-related symptoms aligns with prior reports of diagnostic delays^7^ and likely stems from educational and systemic constraints, including limited consultation time, insufficient diagnostic resources, and inadequate hands-on training.^36^^,^^37^ While most respondents correctly identified HREM as the primary diagnostic tool per the 2024 United European Gastroenterology Achalasia Guidelines,^38^ 20.3% remained unfamiliar with it, indicating a critical shortfall in clinical adoption. The HREM access varied by institutional level, though factors such as resource allocation and administrative constraints were not assessed.

Specialty-based disparities highlight uneven expertise: nearly all gastroenterologists demonstrated adequate knowledge, whereas non-gastroenterologists showed lower familiarity and diagnostic confidence. These differences likely reflect clinical exposure rather than inherent specialty-related capability, though causality cannot be inferred. Consistent with regional and international surveys, non-specialist physicians often exhibit limited knowledge and confidence; for instance, surveys in Egypt and across Asia identified substantial gaps in recognizing and diagnosing motility disorders.^14^^,^^17^

High-resolution esophageal manometry access remains a persistent structural challenge, with tertiary-level physicians reporting more barriers despite higher knowledge—likely reflecting referral burden—while primary care physicians often lack awareness of its availability; across hospital levels, internal medicine physicians demonstrate greater achalasia knowledge and more proactive diagnostic behavior than surgeons, consistent with prior reports of limited exposure to esophageal motility training in surgical and general medicine residency programs.^15^ Addressing these specialty-specific disparities requires integrating structured esophageal motility curricula into both surgical and internal medicine training and fostering interdisciplinary collaboration to enhance diagnostic proficiency and optimize patient care.^38^^-^^41^

Despite relatively high treatment knowledge, fewer than half of physicians reported formal achalasia training, with no variation across hospital levels, reflecting inconsistent educational implementation. These gaps likely drive heterogeneity in knowledge, confidence, and referral practices, consistent with evidence that expertise in rare diseases depends on specialty, training, and institutional context.^35^^,^^36^^,^^42^^,^^43^ Although the cross-sectional, self-reported design limits causal inference, persistent gaps across lower-tier institutions and gender- or institution-related disparities highlight the need for coordinated multidisciplinary education and equitable access to diagnostic resources.

This study has several strengths, including a large and heterogeneous physician sample across specialties and hospital levels, which enhances representativeness. Although the questionnaire was not previously validated, it was developed through a systematic review and refined with expert input, supporting its content validity and improving the interpretability of the results.

This study has several limitations. Self-reported data may entail recall and social desirability bias, and convenience sampling with modest response rates may favor academically oriented participants. Variability in institutional resources and specialty distribution may also affect findings, and reported practices were not validated against clinical records. The cross-sectional design precludes causal inference, and exploratory chi-square tests without correction increase the risk of chance associations. Although primary outcomes were modeled using multivariable logistic regression, subgroup analyses were limited by sample size. Nonetheless, the study provides valuable insights into gaps and educational needs in achalasia care.

In conclusion, this nationwide study provides the first comprehensive assessment of physician knowledge, diagnostic behavior, and referral practices for achalasia in Türkiye. Findings reveal substantial heterogeneity across specialties, hospital levels, and physician characteristics, with persistent gaps in practical competence, HREM familiarity, and access to diagnostic resources. Improving outcomes will require targeted education and structured interventions to strengthen diagnostic infrastructure and referral pathways, addressing both systemic and specialty-specific barriers to support timely recognition and management nationwide.

## Supplementary Materials

Supplementary Material

## Figures and Tables

**Table 1.** Demographic and Professional Characteristics of Participants (n = 675)

Variable	Category	n	%
Age distribution	24-34 years	186	27.6
	35-44 years	264	39.1
	45-54 years	154	22.8
	≥55 years	71	10.5
	Total under 45 years	450	66.7
Gender	Female	388	57.5
	Male	287	42.5
Gastroenterology specialty	Yes	66	9.8
	No	609	90.2
Internal medicine	Yes	264	39.1
Internal medicine subspecialties	Yes	86	12.7
Medical science branch	Internal Medicine Disciplines	605	89.6
	Primary care physicians	83	12.3
	Surgical sciences	66	9.8
	Basic medical sciences	4	0.6
Hospital level	Primary care center	83	12.3
	Secondary Level Hospital	178	26.4
	Tertiary—Training and Research Hospital	177	26.2
	Tertiary—University Hospital	237	35.1

“Primary Care Physicians” refer to general practitioners. “Internal Medicine Disciplines” include internal medicine, emergency medicine, family medicine, neurology, psychiatry, dermatology, physical medicine and rehabilitation, and pediatrics. “Internal Medicine Subspecialties” refer to post-residency fields such as gastroenterology, nephrology, oncology, hematology, rheumatology, and geriatrics. “Surgical sciences” comprise general surgery, obstetrics and gynecology, orthopedics, urology, otorhinolaryngology, neurosurgery, ophthalmology, and cardiovascular surgery. “Basic Medical Sciences” represent preclinical academic fields such as anatomy, physiology, biochemistry, microbiology, and pathology.

**Table 2.** Knowledge and Awareness of Achalasia Among Participants (n = 675)

Variable	Category	n	%
Knowledge of achalasia	Sufficient knowledge	509	75.4
	Partial knowledge	159	23.6
	No knowledge	7	1.0
Frequency of inquiry about achalasia symptoms	Never	141	20.9
	Rarely	214	31.7
	Sometimes	173	25.6
	Frequently	147	21.8
Knowledge regarding incorrect diagnostic methods	Incorrect or unsure (CT/MRI as diagnostic)	212	31.3
Self-Reported knowledge deficiency	Insufficient knowledge	527	78.1
	Sufficient knowledge	148	21.9
Knowledge of HREM	Knowledgeable	538	79.7
	Not knowledgeable	137	20.3
Patient referral for achalasia	Never referred	378	56.0
	Referred at least 1 patient	297	44.0
Accessibility of HREM	Easily accessible	11	1.6
	Difficult to access	401	59.4
	Inaccessible	38	5.6
	Unsure	225	33.3
Achalasia training	Received formal training	305	45.2
	Partial training	336	49.8
	No training	34	5.0
Knowledge of achalasia treatment	Knowledgeable	583	86.4
	Not knowledgeable	92	13.6

CT, computed tomography; HREM, high-resolution esophageal manometry; MRI; magnetic resonance imaging.

**Table 3.** Logistic Regression Analysis of Factors Associated with Knowledge of Achalasia and High-resolution Esophageal Manometry

Variable	OR	95% CI	*P*
Achalasia knowledge			
Gender (Male vs. female)	1.63	1.10-2.41	.015
Specialty (Gastroenterology vs. other)	5.73	1.75-18.83	.004
Primary field (Internal vs. surgical)	1.87	1.06-3.30	.032
Hospital (Overall)	–	–	.034
Primary	1.00	Reference	–
Secondary vs. primary	1.27	0.72-2.25	.402
Tertiary T&R vs. primary	2.09	1.16-3.76	.014
Tertiary Univ. vs. primary	1.87	1.08-3.26	.026
HREM knowledge			
Gender (Male vs. female)	1.46	0.96-2.21	.076
Specialty (Gastroenterology vs. other)	13.30	1.81-97.75	.011
Primary Field (Internal vs. surgical)	2.25	1.25-4.04	.007
Hospital (Overall)	–	–	.003
Primary	1.00	Reference	–
Secondary vs. primary	2.04	1.12-3.71	.020
Tertiary T&R vs. primary	2.49	1.35-4.58	.003
Tertiary Univ. vs. primary	2.09	1.19-3.68	.011

Primary, primary care; secondary, secondary hospital; tertiary T&R, tertiary training and research hospital; tertiary univ., tertiary university hospital; OR, odds ratio; *p*, significance level. Logistic regression analysis was conducted to identify factors associated with knowledge of Achalasia and high-resolution manometry (HREM) among participants. Regarding Achalasia knowledge, male gender, gastroenterology specialty, and internal medicine as primary field were associated with higher likelihood of knowledge. Hospital level was also significant overall, with third-level teaching & research and university hospitals showing higher odds compared to first-level hospitals. For HREM knowledge, gastroenterology specialists had markedly higher odds, and internal medicine and higher hospital levels were also positively associated. Gender showed a borderline significant effect. These findings indicate that specialty and hospital level are key determinants for both Achalasia and HREM knowledge.
